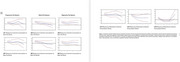# Evaluating the Impact of Alcohol on Dementia: Evidence from 13 Developing Countries

**DOI:** 10.1002/alz70858_096932

**Published:** 2025-12-24

**Authors:** Cyprian M Mostert

**Affiliations:** ^1^ Global Brain Health Institute, Trinity College Dublin, University of Dublin, Ireland

## Abstract

**Background:**

The 2024 Lancet Commission on Dementia Prevention identified alcohol consumption as a risk factor contributing to a global increase in dementia cases by one percent.

**Method:**

We used a panel VAR model to analyze the impact of alcohol consumption on dementia prevalence in 13 developing countries with different alcohol tax systems.

**Result:**

In countries with progressive alcohol tax systems, an increase in taxes on beer and spirits leads to a 10 percent reduction in per capita alcohol consumption. This decline corresponds to a 4 percent reduction in dementia prevalence within four years. In countries with hybrid alcohol tax systems, a combination of alcohol bans and tax increases results in a 12 percent decrease in per capita alcohol consumption, which corresponds to a 30 percent reduction in dementia prevalence within four years. Conversely, in countries with regressive alcohol tax systems, taxes on spirits or beer do not decrease per capita alcohol consumption. In this group of countries, the rise in alcohol consumption per capita leads to an 8 percent increase in dementia prevalence.

**Conclusion:**

The commission's claim that alcohol control could lead to a one percent improvement in global dementia rates is questionable. Countries with progressive and hybrid alcohol tax systems show outcomes that exceed this one percent threshold.